# Prevalence and Diversity of Avian Influenza Virus Hemagglutinin Sero-Subtypes in Poultry and Wild Birds in Bangladesh

**DOI:** 10.3390/vetsci7020073

**Published:** 2020-06-01

**Authors:** Mohammad M. Hassan, Mohamed E. El Zowalaty, Ariful Islam, Shahneaz A. Khan, Md. K. Rahman, Josef D. Järhult, Md. A. Hoque

**Affiliations:** 1 Faculty of Veterinary Medicine, Chattogram Veterinary and Animal Sciences University, Chattogram 4225, Bangladesh; arif@ecohealthalliance.org (A.I.); shahneazbat@gmail.com (S.A.K.); md.hoque@my.jcu.edu.au (M.A.H.); 2 Department of Medical Biochemistry and Microbiology, Zoonosis Science Center, Uppsala University, SE-75 123 Uppsala, Sweden; 3 St. Jude Center of Excellence for Influenza Research and Surveillance, Division of Virology, Department of Infectious Diseases, St Jude Children’s Hospital, Memphis, TN 38105, USA; 4 Centre for Integrative Ecology, School of Life and Environmental Science, Deakin University, Geelong Campus, Geelong, VIC 3125, Australia; 5 EcoHealth Alliance, New York, NY 10001-2320, USA; kaisarrahman@ecohealthalliance.org; 6 Department of Medical Sciences, Zoonosis Science Center, Uppsala University, SE-752 36 Uppsala, Sweden; Josef.Jarhult@medsci.uu.se

**Keywords:** avian influenza, serotype, diversity, domestic birds, wild birds, poultry, ELISA, serology, hemagglutitnin, antibodies, prevalence

## Abstract

Highly pathogenic avian influenza H5 viruses have pandemic potential, cause significant economic losses and are of veterinary and public health concerns. This study aimed to investigate the distribution and diversity of hemagglutinin (HA) subtypes of avian influenza virus (AIV) in poultry and wild birds in Bangladesh. We conducted an avian influenza sero-surveillance in wild and domestic birds in wetlands of Chattogram and Sylhet in the winter seasons 2012–2014. We tested serum samples using a competitive enzyme-linked immunosorbent assay (c-ELISA), and randomly selected positive serum samples (170 of 942) were tested using hemagglutination inhibition (HI) to detect antibodies against the 16 different HA sero-subtypes. All AIV sero–subtypes except H7, H11, H14 and H15 were identified in the present study, with H5 and H9 dominating over other subtypes, regardless of the bird species. The diversity of HA sero-subtypes within groups ranged from 3 (in household chickens) to 10 (in migratory birds). The prevalence of the H5 sero-subtype was 76.3% (29/38) in nomadic ducks, 71.4% (5/7) in household chicken, 66.7% (24/36) in resident wild birds, 65.9% (27/41) in migratory birds and 61.7% (29/47) in household ducks. Moreover, the H9 sero-subtype was common in migratory birds (56%; 23/41), followed by 38.3% (18/47) in household ducks, 36.8% (14/38) in nomadic ducks, 30.6% (11/66) in resident wild birds and 28.5% (2/7) in household chickens. H1, H4 and H6 sero-subtypes were the most common sero-subtypes (80%; 8/10, 70%; 7/10 and 70%; 7/10, respectively) in migratory birds in 2012, H9 in resident wild birds (83.3%; 5/6) and H2 in nomadic ducks (73.9%; 17/23) in 2013, and the H5 sero-subtype in all types of birds (50% to 100%) in 2014. The present study demonstrates that a high diversity of HA subtypes circulated in diverse bird species in Bangladesh, and this broad range of AIV hosts may increase the probability of AIVs’ reassortment and may enhance the emergence of novel AIV strains. A continued surveillance for AIV at targeted domestic–wild bird interfaces is recommended to understand the ecology and evolution of AIVs.

## 1. Introduction

Avian influenza viruses (AIVs) are of great significance to public health due to their potential to cause influenza epidemics and pandemics. Influenza is caused by negative sense single-stranded RNA viruses which belong to the *Orthomyxoviridae* family [1]. *Orthomyxoviridae* includes seven genera, of which only influenza A, B, C and D cause influenza in vertebrates, whereas the influenza A virus genus is mostly known to infect wild and domestic birds [2]. High pathogenicity avian influenza (HPAI) H5N1 is a continuous major pathogen causing high mortality in a variety of avian species and is capable of causing sporadic human infections and mortality [3]. HPAI H5 viruses continue to be a devastating threat to the poultry industry and an incipient threat to humans with a low level of infection. Since 1997, the HPAI H5 virus has continued to spread and evolve. Since 2004, the HPAI H5 virus has spread to many countries worldwide and has been responsible for the destruction of many millions of birds. Wild birds are often blamed for the dispersal of AIVs including HPAI H5 viruses, but definitive proof is often lacking. To date, all human influenza pandemics are associated with H1, H2 and H3 subtypes, but H5, H6, H7, H9 and H10 can also cross the species barrier and infect mammalian species including humans by either antigenic drift or viral strain re-assortment with a human strain as the antigenic shift [4,5]. The outbreak of HPAI has occurred in over 60 countries [6], though it was first identified in China in 1996, and the influenza A/Goose/Guangdong/1/96 (H5N1) isolate is regarded as the ancestor of the present zoonotic H5N1 virus evolution of H5N1 AIVs across Asia [7].

Bangladesh first experienced the outbreak of HPAI in poultry in March 2007. Since then, several outbreaks have been reported in different poultry sectors which were affected by a massive economic loss [8]. There is a huge influx of migratory birds of about 60 different species and approximately 50,000 individual birds [9] which are in close proximity to and mixing with resident wild birds during winter seasons in the major wetlands of Hakaluki and Tanguar haors (wetland ecosystems) of the Sylhet division in Bangladesh. The water bodies in Bangladesh might have played a significant role in the epidemiology and ecology of HPAI (H5N1) outbreaks in local poultry through mixing with domestic waterfowl [10]. However, this statement was not well proven as the prevalence estimates of AIV in domestic and migratory birds were identical in Bangladesh [11], which justifies conducting the present study. 

Domestic household ducks shed influenza viruses asymptomatically and have an important role in the transmission of AIVs (low-pathogenic avian influenza (LPAI) and HPAI) to other susceptible poultry species such as domestic chickens when they intermingle in a common place [12,13]. Nomadic domestic ducks were considered as a connector species between wild or migratory birds and domestic chickens, which may be the key species in the spreading of AIVs. Previous H5N1 outbreaks in domestic poultry have been provoked by nomadic (free-range) ducks in Thailand [14]. The large-scale trading of poultry and poultry products, the presence of live bird wet markets, and wild and migratory bird movements are drivers for spreading AIVs in several countries in Asia including Bangladesh [15,16], China, Hong Kong and South Korea. In China, LPAI H7N9 viruses were transmitted to chicken in live bird markets from domestic ducks and then transmitted to humans [17]. In late 2013 and early 2014 in Jiangxi, China, cases of human infections with H10N8 virus were identified, and a similar serotype was also detected in live bird markets [18].

Since the shedding period of AIVs is varied [19,20,21] in many species of migratory and resident wild birds [22,23], virological or molecular studies do not capture the full temporal picture of AIV circulation and ecology. The viral shedding period in chickens and ducks is longer (up to 2 weeks), but still limited in time [21]. Therefore, a sero-epidemiological study of avian influenza (AI) is justified to explore the exposure status of AIVs belonging to a broad range of hemagglutinin (HA) subtypes. Hence, the current study was conducted to understand the distribution and diversity of HA sero-subtypes of AI in different bird species including domestic and wild birds in Bangladesh.

## 2. Materials and Methods

### 2.1. Ethical Approval

The capturing of free-living and domestic birds was approved by the Bangladesh Forest Department, The People’s Republic of Bangladesh (Reference number: WASU/FAO/PSWMID-6/2012/58). The handling and sampling of birds were approved by the Animal Experimentation Ethics Committee of the Chattogram Veterinary and Animal Sciences University, Bangladesh (Reference number: CVASU/Dir (R and E) AEEC/2015/02). Free living birds were released into the wild after sampling. All efforts were made to minimize animal suffering throughout the research project.

### 2.2. Bird Capture

**Figure 1:**
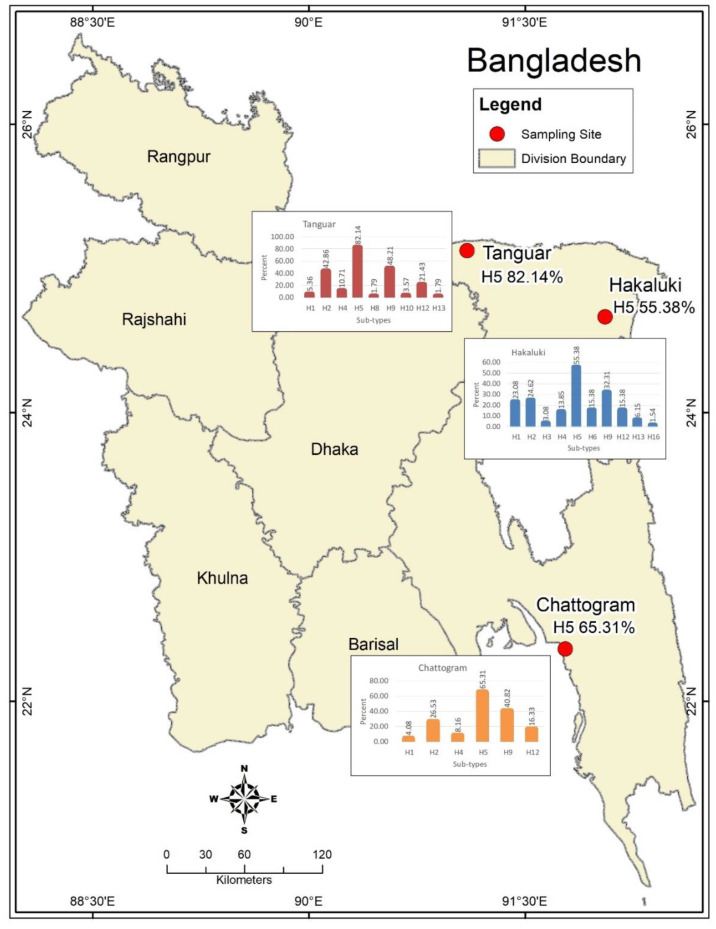
A map of Bangladesh showing the study areas and the spatial distribution of AIV sero-subtypes. The map was plotted using ArcMap, version 10.2, Environmental Systems Research Institute, Redlands, CA, USA.

A wide variety of migratory (Ferruginous duck, Gadwall, Lesser whistling duck, Little grebe, Northern pintail, Ruddy shelduck, Tufted duck, etc.) and resident (Asian pied starling, Brown-headed gull, House crow, House sparrow) wild birds, along with domestic poultry from major wetlands in Bangladesh (Figure 1, Supplementary Table S1), were captured during the winter season (January–March) over three consecutive years, 2012–2014. 

Migratory wild birds visiting the Hakaluki (Moulavibazar) and Tanguar Hoar (Sunamganj) wetlands were sampled. Bangladeshi resident wild birds at roosting sites in the area of Hakaluki and Tanguar haors and Chattogram were sampled as well. Domestic birds such as household chickens, ducks and nomadic ducks (a group of free range ducks raised at the bank of waterbodies) were randomly sampled, at the rate of one bird per household (flock size: five to ten birds for chickens; four to ten birds for ducks) and five birds per nomadic flock (200 to 1000 birds per flock). All resident and migratory wild birds were caught using mist nets under the permission of the Wildlife Division of the Department of Forestry, Bangladesh.

### 2.3. Sample Collection, Competitive Enzyme-Linked Immunosorbent Assay and Haemagglutination Inhibition Test 

Blood samples were collected from each individual bird as previously described [11]. Serum samples were initially evaluated using a competitive enzyme-linked immunosorbent assay (c-ELISA) (AI Multi-screen kit; IDEXX Laboratory Inc., Westbrook, ME, USA, lot: AM688) according to the manufacturer’s instructions [24], and samples were considered as reactive when a sample had a mean value of 40% inhibition or more. Of the 2985 serum samples tested, 942 were c-ELISA AI antibody reactive. From the c-ELISA reactive samples, 170 (41 migratory birds, 36 resident wild birds, 38 nomadic ducks, 47 household ducks and 7 household chickens) were randomly selected for a haemagglutination inhibition (HI) test. The HI test used a panel of inactivated AIV HA antigens (H1–H16) for this study, provided by the Australian Animal Health Laboratory (AAHL), to determine the distribution of AIV HA sero-subtypes infecting birds as previously described [24,25]. Sera showing inhibition at dilutions of 1:16 or greater against four haemagglutination units of AIV antigen were considered reactive for the antibody of migratory or resident aquatic wild birds as was previously reported [24], whereas sera showing inhibition of 1:64 or more against four haemagglutination units of AIV antigen were considered reactive for domestic chickens and ducks as was previously reported [25].

### 2.4. Statistical Analysis

Data were entered into Microsoft Excel 2010 and checked for integrity before exporting to STATA/IC 13 (Stata Corp, College Station, TX, USA). A descriptive statistical analysis of the HI results was performed. The AIV serological distribution was shown by subtypes, bird groups, study sites and year. The results were expressed in frequency numbers and percentages in tables and graphs. 

## 3. Results

The distribution of AIV HA serotypes was presented as the diversity of subtypes and ranged from three (H5, H9 and H12) in household chickens to ten (H1, H2, H3, H4, H5, H6, H9, H12, H13 and H16) in migratory birds. Moreover, we also detected five subtypes (H1, H2, H5, H9 and H12) in resident wild birds, 7 (H1, H2, H4, H5, H9, H10 and H12) in nomadic ducks and eight (H1, H2, H4, H5, H8, H9, H12 and H13) in household ducks. In general, the H5 serotype was highly prevalent in all study locations (Figure 1) and across all bird groups, followed by H9, H2, H12 and other serotypes (Table 1, Supplementary Table S1). 

In migratory birds, the frequency of the H5 serotype was found to be the most prevalent (65.9%; 27/41) followed by H9 (56%; 23/41), H1 (41.5%; 17/41), H12 (41.5%; 17/41) and H2 (34.2%; 14/41). In resident wild birds, the distribution of HA serotypes was 66.7% (24/36) H5, 30.6% (11/36) H9 and 25% (9/36) H2. In domestic birds, the HA serotype frequencies were 61.7% for H5 (29/47) and 38.3% for H9 (18/47) in household ducks, 76.3% for H5 (29/38), 55.3% for H2 (21/38) and 36.8% for H9 (14/38) in nomadic ducks, and 71.4% for H5 (5/7) and 28.5% for H9 (2/7) in household chickens (Table 1).

The grouping of the HA sero-subtype distribution by the sampling year resulted in the finding that the dominant subtype in 2012 was H1 (80%; 8/10) in migratory birds, followed by H4 (66.6%; 4/6) in household ducks and H5 (64.3%; 18/28) in resident wild birds. In 2013, H9 (83.3%; 5/6) and H5 (66.6%; 4/6) were the most common sero-subtypes in resident wild birds, followed by H2 (73.9%; 17/23) and H5 (69.6%; 16/23) in nomadic ducks. In 2014, H5 (100%; 15/15) and H9 (66.7%; 10/15) were the most common sero-subtypes in migratory birds, followed by H5 (100%; 2/2) in resident wild birds, H5 (86.7%; 13/15) in nomadic ducks and H5 (64.9%; 24/37) and H9 (40.5%; 15/37) in household ducks (Table 2).

The grouping of the HA sero-subtype distribution by the sampling site demonstrated that both hoars were the most infected hub by H5 and H9 in comparison to other sites. The following was found: 39.1% (9/23) in Hakaluki haor vs. 100% (17/17) in Tanguar haor vs. 100% (1/1) in Chattogram for H5 and 52.2% (12/23) in Hakaluki haor vs. 58.8% (10/17) in Tanguar haor vs. 100% (1/1) in Chattogram for H9 in migratory birds; 100% (1/1) in Hakaluki haor vs. 100% (3/3) in Tanguar haor vs. 62.5% (20/32) in Chattogram for H5 and 0% (0/1) in Hakaluki haor vs. 33.3% (1/3) in Tanguar haor vs. 31.3% (10/32) in Chattogram for H9 in resident wild birds; 86.7% (13/15) in Hakaluki haor vs. 69.6% (16/23) in Tanguar haor vs. 0% in Chattogram for H5 and 33.3% (5/15) in Hakaluki haor vs. 39.1% (9/23) in Tanguar haor vs. 0% in Chattogram for H9 in nomadic ducks; 50% (11/22) in Hakaluki haor vs. 76.9% (10/13) in Tanguar haor vs. 61.5% (8/13) in Chattogram for H5 and 18.2% (4/22) in Hakaluki haor vs. 53.8% (7/13) in Tanguar haor vs. 53.8% (7/13) in Chattogram for H9 in household ducks. It was found that sero-subtypes H1, H2, H3, H4, H6, H8, H10, H12, H13 and H16 were detected across the study sites in different bird types (Table 3).

## 4. Discussion

In the present study, multiple AIV HA sero-subtypes were detected in migratory birds, household ducks and nomadic ducks [26], which corroborates that waterfowl are the natural reservoir for a wide range of AIVs [7,8,9,10,11,12]. The highest HA sero-subtype diversity was observed in migratory birds, consistent with the fact that AIV diversity is driven by this group, which introduces new AIVs through migration. The finding that eight different HA serotypes circulated in resident wild birds in Bangladesh further suggested that resident wild birds may have been infected with AIVs from aquatic birds (migratory birds and household ducks) when they roosted at the same roosting sites of wetlands of Hakaluki and Tanguar Hoars and Chattogram. The resident birds are suggested to have played a role as accidental hosts of AIVs. The presence of multiple HA sero-subtypes in resident birds is supported by several previous studies from different parts of the world [27,28]. The detection of fewer numbers of HA sero-subtypes in domestic chickens, which is similar to other studies [5], is consistent with the fact that these birds are not yet identified as enzootic or natural hosts for a wide range of AIVs. However, the low number of sera from household chickens which were subjected to the serotype-specific analysis may lead to an underestimation of diversity which mandates further studies. 

Irrespective of bird groups, H5 and H9 HA sero-subtypes were the most commonly detected sero-subtypes, which suggested that the birds at the study areas were in close contact (e.g., common roosting places), allowing the transmission of AIVs. This possibility is strongly supported by several reports [29,30,31]. The presence of the H5 sero-subtype in nomadic ducks may be explained by their mixing with migratory birds in the wetland environment. Migratory birds, resident wild birds and free-ranging domestic birds, the type of farming system [32], and the nature of wetland ecosystems are key factors in maintaining the circulation and spread of different AIV HA subtypes in Bangladesh.

Along with H5 subtypes, the finding of H9 sero-subtypes in domestic birds in the present study is similar to a previous report [33]. The potential for humans to be infected with AIVs is due to the fact that chickens and ducks in many households are mixed together in night shelters at human dwellings. AI H5 and H9 subtypes are the most well-known virus subtypes to cause human infections [34]. The movement of resident wild birds between different places in live bird markets and the abundance of live bird markets pose a risk of interspecies transmission of AIVs [26], and represent a serious threat for human health due to the emergence of AIVs with pandemic potential. 

The diversity of different subtypes varied among different wetlands. It was found that H2, H5 and H9 sero-subtypes were the most prevalent in Tanguar Hoar, whereas H1, H9, H6 and H12 sero-subtypes were predominantly found in Hakaluki Hoar. In Chattogram, H5, H9 and H12 sero-subtypes were frequently found among different bird types. These disparities of HA sero-subtypes can be partially explained by the variation of sampling location, mixing of bird types and the susceptibility to AIVs. The discrepancy is supported by a previous study [35]. The common detection of H5 and H9 sero-subtypes in all sampling sites suggested that these subtypes are established in Bangladesh, whereas other subtypes may be more transient and potentially introduced by exotic migratory birds. AIV antibodies corresponding to multiple HA subtypes were frequently detected in individual birds in the present study. Moreover, previous studies reported more than one AIV subtype infecting individual wild ducks [36,37]. These previous results, along with the sero-typing results in the current study, indicate that wild birds can be infected with more than one AIV subtype throughout their lives. The co-infection with more than one AIV subtype further increases the risk of virus reassortment. In the present study, multiple sero-subtypes were found in a single sample, which indicated that bird groups were exposed to multiple subtypes during their lifetime. However, the potential for cross-contamination of HA serotypes in the HI testing of the serum samples could have occurred, although the high cut-off HI titre was used to confirm the sero-positivity of different HA serotypes. Sero-surveillance offers estimates of the immunity of populations against preventable diseases such as AIV, and it is important to evaluate the immunity level of populations against certain diseases [38]. In addition, the correlation between the strength of an HI result and the possible time since a bird was infected is not straightforward. In general, HI titres decrease over time, but sometimes sub-clinical infection raises the antibody titre again, and reservoir species may also have some degree of immunity but still infect other susceptible species. The results of the present study therefore suggest that AIVs could spill over to resident or domestic birds from migratory birds and potentially spill back to migratory birds (for some HA sero-subtypes) from the resident or domestic birds. Therefore, it is likely that the AIV ecology in birds in Bangladesh allows for evolution (e.g., through reassortment), and it is important to identify warning signs which may point to the emergence of a potentially virulent AIV strain in domestic birds and highly transmissible AIVs to humans [39]. Thus, further studies are required to investigate the direction and extent of AIV exchange between wild and domestic birds. In addition, future molecular studies are required to determine the evolutionary patterns of AIVs in the country. 

## 5. Conclusions

Avian influenza (AI) has pandemic potential, causes significant economic losses in poultry in Bangladesh and is of great concern to public health. The current study underlines the importance of sero-surveillance in wild and domestic birds. In the present study, the diversity of HA sero sub-types ranged from minimum in household chickens to maximum in migratory birds. In general, the prevalence of all HA sero-subtypes was higher in migratory birds in comparison with other bird types. Little variation of HA subtype patterns was observed in different sites and over the sampling years. Furthermore, the prevalence of the H5 sero-subtype was high in all locations. The findings of the present study showed a high diversity of HA subtypes circulating in diverse wild bird species (both migratory and resident). The findings also suggest expanding the current host range of the AIV reservoir, which may increase the probability of AIV reassortment and the emergence of novel strains. Continuous surveillance efforts for AIVs are recommended to fully understand the ecology and evolutionary patterns of HA subtypes in wild and domestic birds.

## Figures and Tables

**Table 1:** Matrix of bird type and avian influenza haemagglutinin (HA) serotype in Bangladesh.

HA Subtype	H1	H2	H3	H4	H5	H6	H7	H8	H9	H10	H11	H12	H13	H14	H15	H16
Migratory bird (*n* = 41, N = 170)	●●●●●●●●●●●●●●●●●	●●●●●●●●●●●●●●	●●	●●●●●●●●●●●●●	●●●●●●●●●●●●●●●●●●●●●●●●●●●	●●●●●●●●●●			●●●●●●●●●●●●●●●●●●●●●●●			●●●●●●●●●●●●●●●●●	●●●●			●
Resident wild bird (*n* = 36, N = 170)	●	●●●●●●●●●			●●●●●●●●●●●●●●●●●●●●●●●				●●●●●●●●●●●			●●				
Nomadic duck (*n* = 38, N = 170)	●	●●●●●●●●●●●●●●●●●●●●●		●●	●●●●●●●●●●●●●●●●●●●●●●●●●●●●●				●●●●●●●●●●●●●●	●●		●●●●●●				
Household duck (*n* = 47, N = 170)	●	●●●●●●●●●		●●●●	●●●●●●●●●●●●●●●●●●●●●●●●●●●●●			●	●●●●●●●●●●●●●●●●●●			●●●●	●			
Household chicken (*n* = 7, N = 170)					●●●●●				●●			●				

*n* = number of c-ELISA reactors tested for HA serotype; N = Total number of c-ELISA positive serum samples used for HA serotype. •: frequency number of serotypes (multiple serotypes in individual samples were counted individually).

**Table 2:** Year versus haemagglutinin (HA) serotypes for different groups of birds in Bangladesh.

AIV Subtype	2012 (January–March)					2013 (January–March)					2014 (January–March)				
	MB	RW	ND	HD	HC	MB	RW	ND	HD	HC	MB	RW	ND	HD	HC
	(N = 10)	(N = 28)	(N = 0)	(N = 6)	(N = 0)	(N = 16)	(N = 6)	(N = 23)	(N = 5)	(N = 3)	(N = 15)	(N = 2)	(N = 15)	(N = 37)	(N = 4)
	*n* (%)	*n* (%)	*n* (%)	*n* (%)	*n* (%)	*n* (%)	*n* (%)	*n* (%)	*n* (%)	*n* (%)	*n* (%)	*n* (%)	*n* (%)	*n* (%)	*n* (%)
H1	8 (80)	0 (0)	0 (0)	0 (0)	0 (0)	7 (43.8)	1 (16.7)	0 (0)	0 (0)	0 (0)	2 (13.3)	0 (0)	1 (6.7)	1 (2.7)	0 (0)
H2	3 (30)	8 (28.6)	0 (0)	2 (33.3)	0 (0)	6 (37.5)	1 (16.7)	17 (73.9)	2 (40)	0 (0)	5 (33.3)	0 (0)	4 (26.7)	5 (13.5)	0 (0)
H3	2 (20)	0 (0)	0 (0)	0 (0)	0 (0)	0 (0)	0 (0)	0 (0)	0 (0)	0 (0)	0 (0)	0 (0)	0 (0)	0 (0)	0 (0)
H4	7 (70)	0 (0)	0 (0)	4 (66.6)	0 (0)	3 (18.8)	0 (0)	2 (8.7)	0 (0)	0 (0)	3 (20)	0 (0)	0 (0)	0 (0)	0 (0)
H5	2 (20)	18 (64.3)	0 (0)	3 (50)	0 (0)	10	4 (66.7)	16 (69.6)	2 (40)	3 (100)	15 (100)	2 (100)	13 (86.7)	24 (64.9)	2 (50)
H6	7 (70)	0 (0)	0 (0)	0 (0)	0 (0)	3 (18.8)	0 (0)	0 (0)	0 (0)	0 (0)	0 (0)	0 (0)	0 (0)	0 (0)	0 (0)
H7	0 (0)	0 (0)	0 (0)	0 (0)	0 (0)	0 (0)	0 (0)	0 (0)	0 (0)	0 (0)	0 (0)	0 (0)	0 (0)	0 (0)	0 (0)
H8	0 (0)	0 (0)	0 (0)	0 (0)	0 (0)	0 (0)	0 (0)	0 (0)	1 (20)	0 (0)	0 (0)	0 (0)	0 (0)	0 (0)	0 (0)
H9	6 (60)	6 (21.4)	0 (0)	2 (33.3)	0 (0)	7 (43.8)	5 (83.3)	9 (39.1)	1 (20)	2 (66.7)	10 (66.7)	0 (0)	5 (33.3)	15 (40.5)	0 (0)
H10	0 (0)	0 (0)	0 (0)	0 (0)	0 (0)	0 (0)	0 (0)	2 (8.7)	0 (0)	0 (0)	0 (0)	0 (0)	0 (0)	0 (0)	0 (0)
H11	0 (0)	0 (0)	0 (0)	0 (0)	0 (0)	0 (0)	0 (0)	0 (0)	0 (0)	0 (0)	0 (0)	0 (0)	0 (0)	0 (0)	0 (0)
H12	5 (50)	2 (7.1)	0 (0)	2 (33.3)	0 (0)	6 (37.5)	0 (0)	5 (21.7)	0 (0)	1 (33.3)	6 (40)	0 (0)	1 (6.7)	2 (5.4)	0 (0)
H13	3 (30)	0 (0)	0 (0)	0 (0)	0 (0)	1 (6.3)	0 (0)	0 (0)	1 (20)	0 (0)	0 (0)	0 (0)	0 (0)	0 (0)	0 (0)
H14	0 (0)	0 (0)	0 (0)	0 (0)	0 (0)	0 (0)	0 (0)	0 (0)	0 (0)	0 (0)	0 (0)	0 (0)	0 (0)	0 (0)	0 (0)
H15	0 (0)	0 (0)	0 (0)	0 (0)	0 (0)	0 (0)	0 (0)	0 (0)	0 (0)	0 (0)	0 (0)	0 (0)	0 (0)	0 (0)	0 (0)
H16	1 (10)	0 (0)	0 (0)	0 (0)	0 (0)	0 (0)	0 (0)	0 (0)	0 (0)	0 (0)	0 (0)	0 (0)	0 (0)	0 (0)	0 (0)

MB: Migratory bird; RW: Resident wild bird; ND: Nomadic duck; HD: Household duck; HC: Household chicken.

**Table 3:** Area versus haemagglutinin (HA) serotypes for different groups of birds in Bangladesh.

AIV Subtype	Hakaluki Hoar					Tanguar Hoar					Chittagong				
	MB	RW	ND	HD	HC	MB	RW	ND	HD	HC	MB	RW	ND	HD	HC
	(N = 23)	(N = 1)	(N = 15)	(N = 22)	(N = 4)	(N = 17)	(N = 3)	(N = 23)	(N = 13)	(N = 0)	(N = 1)	(N = 32)	(N = 0)	(N = 13)	(N = 3)
	*n* (%)	*n* (%)	*n* (%)	*n* (%)	*n* (%)	*n* (%)	*n* (%)	*n* (%)	*n* (%)	*n* (%)	*n* (%)	*n* (%)	*n* (%)	*n* (%)	*n* (%)
H1	14 (60.9)	0 (0)	1 (6.7)	0 (0)	0 (0)	3 (17.6)	0 (0)	0 (0)	0 (0)	0 (0)	0 (0)	1 (3.1)	0 (0)	1 (7.7)	0 (0)
H2	9 (39.1)	0 (0)	4 (26.7)	3 (13.7)	0 (0)	5 (29.4)	0 (0)	17 (73.9)	2 (15.4)	0 (0)	0 (0)	9 (28.1)	0 (0)	4 (30.8)	0 (0)
H3	2 (8.7)	0 (0)	0 (0)	0 (0)	0 (0)	0 (0)	0 (0)	0 (0)	0 (0)	0 (0)	0 (0)	0 (0)	0 (0)	0 (0)	0 (0)
H4	9 (39.1)	0 (0)	0 (0)	0 (0)	0 (0)	4 (23.5)	0 (0)	2 (8.6)	0 (0)	0 (0)	0 (0)	0 (0)	0 (0)	4 (30.8)	0 (0)
H5	9 (39.1)	1 (100)	13 (86.7)	11 (50)	2 (50)	17 (100)	3 (100)	16 (69.6)	10 (76.9)	0 (0)	1 (100)	20 (62.5)	0 (0)	8 (61.5)	3 (100)
H6	10 (43.5)	0 (0)	0 (0)	0 (0)	0 (0)	0 (0)	0 (0)	0 (0)	0 (0)	0 (0)	0 (0)	0 (0)	0 (0)	0 (0)	0 (0)
H7	0 (0)	0 (0)	0 (0)	0 (0)	0 (0)	0 (0)	0 (0)	0 (0)	0 (0)	0 (0)	0 (0)	0 (0)	0 (0)	0 (0)	0 (0)
H8	0 (0)	0 (0)	0 (0)	0 (0)	0 (0)	0 (0)	0 (0)	0 (0)	1 (7.7)	0 (0)	0 (0)	0 (0)	0 (0)	0 (0)	0 (0)
H9	12 (52.2)	0 (0)	5 (33.3)	4 (18.2)	0 (0)	10 (58.8)	1 (33.3)	9 (39.1)	7 (53.8)	0 (0)	1 (100)	10 (31.3)	0 (0)	7 (53.8)	2 (66.7)
H10	0 (0)	0 (0)	0 (0)	0 (0)	0 (0)	0 (0)	0 (0)	2 (8.7)	0 (0)	0 (0)	0 (0)	0 (0)	0 (0)	0 (0)	0 (0)
H11	0 (0)	0 (0)	0 (0)	0 (0)	0 (0)	0 (0)	0 (0)	0 (0)	0 (0)	0 (0)	0 (0)	0 (0)	0 (0)	0 (0)	0 (0)
H12	9 (39.1)	0 (0)	1 (6.7)	0 (0)	0 (0)	7 (41.2)	0 (0)	5 (21.7)	0 (0)	0 (0)	1 (100)	2 (6.3)	0 (0)	4 (30.8)	1 (33.3)
H13	4 (17.4)	0 (0)	0 (0)	0 (0)	0 (0)	0 (0)	0 (0)	0 (0)	1 (7.7)	0 (0)	0 (0)	0 (0)	0 (0)	0 (0)	0 (0)
H14	0 (0)	0 (0)	0 (0)	0 (0)	0 (0)	0 (0)	0 (0)	0 (0)	0 (0)	0 (0)	0 (0)	0 (0)	0 (0)	0 (0)	0 (0)
H15	0 (0)	0 (0)	0 (0)	0 (0)	0 (0)	0 (0)	0 (0)	0 (0)	0 (0)	0 (0)	0 (0)	0 (0)	0 (0)	0 (0)	0 (0)
H16	1 (4.3)	0 (0)	0 (0)	0 (0)	0 (0)	0 (0)	0 (0)	0 (0)	0 (0)	0 (0)	0 (0)	0 (0)	0 (0)	0 (0)	0 (0)

MB: Migratory bird; RW: Resident wild bird; ND: Nomadic duck; HD: Household duck; HC: Household chicken.

## Supplementary Materials

The following are available online at https://www.mdpi.com/2306-7381/7/2/73/s1, Table S1: metadata of bird samples during avian influenza surveillance in 2012–2014 in Bangladesh.
